# Turn‐On Photocatalysis: Creating Lone‐Pair Donor–Acceptor Bonds in Organic Photosensitizer to Enhance Intersystem Crossing

**DOI:** 10.1002/advs.202100631

**Published:** 2021-08-02

**Authors:** Mingjie Liu, Junnan Liu, Kai Zhou, Jingwen Chen, Qi Sun, Zongbi Bao, Qiwei Yang, Yiwen Yang, Qilong Ren, Zhiguo Zhang

**Affiliations:** ^1^ Key Laboratory of Biomass Chemical Engineering of Ministry of Education College of Chemical and Biological Engineering Zhejiang University Zheda Road 38 Hangzhou 310027 China; ^2^ Institute of Zhejiang University‐Quzhou 78 Jiuhua Boulevard North Quzhou 324000 China

**Keywords:** covalent organic frameworks, intersystem crossing, organic photosensitizer, photocatalysis, singlet oxygen, triazine

## Abstract

There is growing interest in developing triplet photosensitizers in terms of implementing photochemical strategies in synthetic chemistry. However, synthesis of stable triplet organic photosensitizers is nontrivial and often requires the use of heavy atoms. Herein, an alternative strategy is demonstrated to enhance the triplet generation efficiency by implanting lone‐pair donor–acceptor bonds in the conjugated covalent organic frameworks (COFs). This powerful method is validated using COFs that host triazine, a moiety that has been extensively investigated in photocatalysis. Spectroscopic analysis and theoretical calculations reveal substantial improvements in the photoabsorptivity and triple‐state photogeneration efficiency, consistent with catalytic tests concerning industrially relevant sulfide oxidation. These systems represent a promising addition to the rapidly increasing arsenal of synthetic photocatalytic systems.

## Graphical abstract

Stable organic triplet photosensitizers are developed by incorporating the oxidated triazine moiety into conjugated covalent organic frameworks. Greatly improved intersystem crossing processes and triplet generation efficiency are observed after anchoring oxygen atoms at the N ends in triazine, steering the photocatalysis toward the energy transfer pathway.

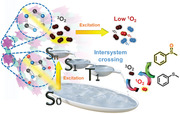

## 1 Introduction

Harvesting solar energy for organic transformation is a practical technology to alleviate the global dependence on fossil fuels, which has also set the stage for innovative molecular synthesis.^[^ ^1^ ^]^ However, control over the evolution of highly reactive intermediates to steer them toward the desired energy transduction pathways remains a long‐standing challenge.^[^ ^2^ ^]^ Triplet excited states (triplets) have been recognized as promising active species in photocatalysis owing to their high energy transport capability and long lifetimes.^[^ ^3^ ^]^ For example, the photosensitized singlet oxygen (^1^O_2_) generated by energy transfer from triplet excited states of sensitizers to molecular dioxygen (O_2_) has played an indispensable role in photoredox reactions.^[^ ^4^ ^]^ However, the development of triplet photosensitizers has not seen much progress. Most of the current systems rely on heavy atoms to promote intersystem crossing (ISC), which often induces dark toxicity and increases the cost of the materials, thus limiting their wide applications.^[^ ^5^ ^]^ To address these challenges, ketone photosensitizers have been developed. However, this type of sensitizer often shows low absorption in the visible region, and the unpaired electron of the oxygen atom of carbanyl group (C═O) in the *n*–π* state is susceptible to hydrogen atom transfer from C—H bonds, resulting in degradation of the sensitizer and side reactions.^[^ ^6^ ^]^ Consequently, the production of stable triplet photosensitizers without the assistance of heavy atoms would further expand their utilization.

Motivated by this purpose, several strategies have been developed to improve the ISC process of photosensitizers, including 1) extending the conjugation of photosensitizers,^[^ ^5^ ^]^ 2) shortening the distance between donor and acceptor,^[^ ^7^ ^]^ and 3) introducing paramagnetic species.^[^ ^8^ ^]^ Accordingly, we wondered if these methods could be merged into one to further boost the ISC process in the resulting materials and the triplet generation efficiency. To this end, herein, we introduce a new concept for the fabrication of high performance triplet photosensitizers by creating lone‐pair donor–acceptor bonds in 2D covalent organic frameworks (2D COFs).^[^ ^9^ ^]^ COFs have flourished as designer platforms that provide access to a wide range of applications relying on their chemical and structural diversity that promise revolution in the fields of optoelectronics,^[^ ^10^ ^]^ environmental remediation,^[^ ^11^ ^]^ sensing,^[^ ^12^ ^]^ and many more.^[^ ^13^ ^]^ The exploration of photocatalysts, in particular, has triggered considerable interest due to the unparalleled advantages of 2D COFs in terms of light utilization.^[^ ^14^ ^]^ The modularity and unique structure of 2D COFs offer the opportunity to program chromophore struts into 2D *π* arrays packed into densely aligned *π* columns, leading to significant electronic overlap. These features not only improve the photoabsorption of chromophores but also promote the transport of excitations. We envisioned that olefin‐linked triazine COFs possess the congenital capability to merge all the merits mentioned above. The COFs offer the superior light‐harvesting ability and more complex energy band structures compared with those of the triazine moiety.^[^ ^15^ ^]^ Moreover, in this state‐of‐the‐art organic molecular photosensitizer, the N ends in triazine could be directly anchored with oxygen atoms to yield an inner molecule lone‐pair donor–acceptor bond of the triazine *N*‐oxide, which exhibits more excellent resistance against deactivation as a result of hydrogen atom transfer.^[^ ^16^ ^]^ Therefore, the resulting materials are expected to facilitate photoinduced energy transfer to improve the ISC process, according to which the excited electrons can be transformed to a degenerate state with a different spin multiplicity, and consequently the accompanied photocatalytic performance.

## 2 Results and Discussion

### 2.1 Material Synthesis and Characterization

To demonstrate the feasibility of this concept, we began our study using 2,4,6‐tris[(E)‐4‐cyanostyryl]‐1,3,5‐triazine (TMTACN), the analog of the strut of olefin‐linked triazine COFs. To introduce O on the N ends of the triazine moiety, TMTACN was treated with ammonium thiosulfate ((NH_4_)_2_S_2_O_8_). The resulting compound structure was unambiguously characterized using nuclear magnetic resonance (NMR) spectroscopy and matrix‐assisted laser desorption/ionization time of flight mass spectrometry (MALDI‐TOF‐MS, Figures S1 and S2, Supporting Information). The signal at *m*/*z* 512 was denoted as the primary signal for the oxidized product, corresponding to the structure with all N ends in the triazine moiety installed with an oxygen atom (TMTACNO). The appearance of the N→O stretching vibration at 1125 cm^–1^ in the Fourier transform infrared (FT‐IR) spectrum of the oxidized product further validated the structure (Figure S3, Supporting Information).^[^ ^17^ ^]^

**Figure 1:**
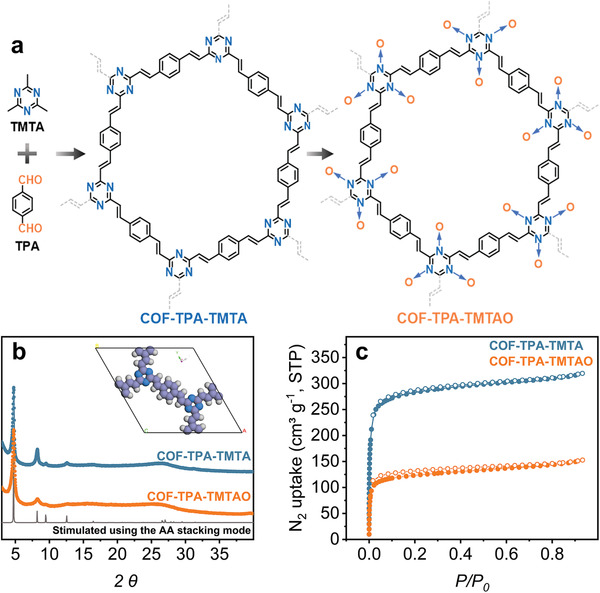
a) Synthetic scheme of COF‐TPA‐TMTA and COF‐TPA‐TMTAO. b) PXRD patterns. Inset: unit cell of the AA stacking mode of COF‐TPA‐TMTA (N, blue; C, grey; H, white). c) N_2_ sorption isotherms for COF‐TPA‐TMTA and COF‐TPA‐TMTAO collected at 77 K.

After proving the efficiency of such a modification, we extended this strategy to its conjugated polymeric counterparts. The olefin‐linked triazine COF (COF‐TPA‐TMTA) synthesized by condensing terephthalaldehyde (TPA) with 2,4,6‐trimethyl‐1,3,5‐triazine (TMTA) was chosen to demonstrate this proof of concept (**Figure** **1**a; Table S1, Supporting Information).^[^ ^15^ ^]^ Successful polymerization and the formation of olefin linkages were confirmed by FT‐IR spectroscopy. The disappearance of the C═O (1700 cm^–1^) and C—H (2930 cm^–1^) stretching bands assigned to TPA and TMTA, respectively, and the emergence of new C═C stretching bands at 1630 and 980 cm^–1^ corresponding to olefin linkages supported the successful condensation of TPA and TMTA (Figure S4, Supporting Information). Furthermore, when compared with the ^13^C NMR spectra of the monomers, those of COF‐TPA‐TMTA exhibited a new resonance at 139 ppm, confirming the formation of a C═C bond (Figure S5, Supporting Information). The crystallinity of COF‐TPA‐TMTA was determined by powder X‐ray diffraction (PXRD, Figure 1b). The experimental pattern matched reasonably with the simulated pattern in a 2D slipped‐AA packing mode in the space group of *P*6/*m*, with 1.8 nm hexagonal channels running along the crystallographic *c*‐axis. The porosity of the COF was demonstrated by N_2_ sorption isotherms collected at 77 K that displayed type I profiles, showing a Brunauer–Emmett–Teller (BET) surface area of 1093 m^2^ g^–1^ (Figure 1c). Fitting the adsorption isotherm using the nonlocal density functional theory (NLDFT) model resulted in a pore size distribution with a maximum centered at 1.8 nm, close to the theoretical value (Figure S6, Supporting Information).

**Figure 2:**
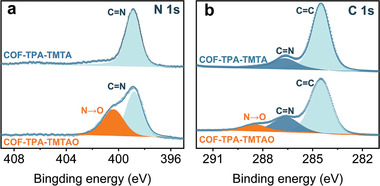
XPS spectra of a) N 1s and b) C 1s for COF‐TPA‐TMTA and COF‐TPA‐TMTAO.

To introduce O on the N ends of triazine, COF‐TPA‐TMTA was reacted with (NH_4_)_2_S_2_O_8_ with the resulting material denoted as COF‐TPA‐TMTAO (Figure 1a and Table S2, Supporting Information). As the reaction proceeded, the initial yellowish reaction mixture gradually changed to orange. No postmodification structural collapse was first suggested by the similar overall morphology of COF‐TPA‐TMTA and COF‐TPA‐TMTAO, as revealed by scanning electron microscopy (SEM) images (Figure S7, Supporting Information). The well‐preserved PXRD pattern confirmed the retained structure (Figure 1b). The successful oxidation was supported by solid‐state ^13^C NMR, FT‐IR, and X‐ray photoelectron spectroscopy (XPS). The solid‐state ^13^C NMR spectra revealed that the peak for C═N in COF‐TPA‐TMTAO shifted slightly to a lower field compared with that of COF‐TPA‐TMTA (Figure S5, Supporting Information). A new stretching peak at 1112 cm^–1^ appeared in the IR spectrum of COF‐TPA‐TMTAO, indicative of the formation of the N→O bond (Figure S8, Supporting Information). The differences in the elemental compositions of the materials before and after oxidation were explored by XPS. The deconvolution of N 1s core‐level spectrum of COF‐TPA‐TMTAO showed two peaks at 398.8 and 400.3 eV, assigned to C═N and C═N→O, respectively (**Figure** **2**a). Concurrently, the appearance of the C 1s peak at the binding energy of 288.5 eV attributable to C═N→O in COF‐TPA‐TMTAO confirmed the occurrence of oxidation (Figure 2b).^[^ ^18^ ^]^ The N→O content was determined by the integral area of C═N→O relative to the overall N content, indicating a functionalization degree of 42%, which was further corroborated by the corresponding result derived from the C 1s spectrum of COF‐TPA‐TMTAO (42%). After oxidation, the BET surface area decreased from 1039 to 484 m^2^ g^–1^, which is reasonable because of the large increase in molecular weight. Thermal gravimetric analysis (TGA) revealed that the resulting COF material was thermally stable up to 200 °C (Figure S9, Supporting Information). The COF materials are light stable, as evidenced by the fact that negligible morphology change was observed after irradiation under the blue LED (40 W) for one week (Figure S7, Supporting Information).

**Figure 3:**
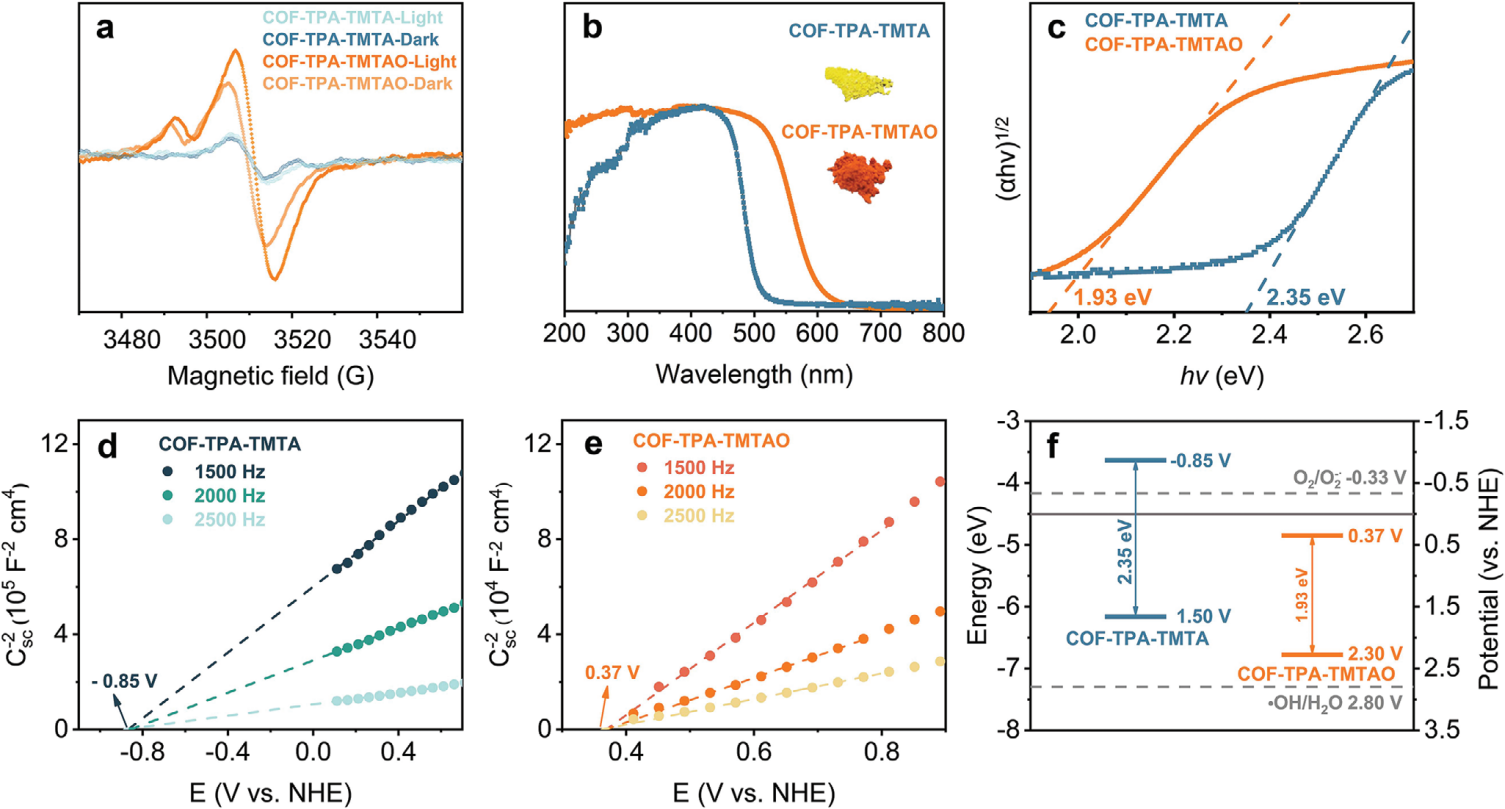
a) EPR spectra of COF‐TPA‐TMTA and COF‐TPA‐TMTAO before and after exposure to light. b) UV–vis spectra (inset: digital photographs). c) Tauc plots. d,e) Mott–Schottky curves. f) The energy band structure.

To investigate the electron properties of the terminal oxygen species in COF‐TPA‐TMTAO, electron paramagnetic resonance (EPR) spectroscopy was conducted. To subtract the background, the EPR signals of COF‐TPA‐TMTA were also collected. The scan time and volume‐normalized EPR data are displayed in **Figure** **3**a. COF‐TPA‐TMTA showed very weak EPR signals, whereas a more vigorous EPR signal intensity was observed for COF‐TPA‐TMTAO, indicating the prevalence of oxygen‐centered radicals. The EPR signal increased upon light irradiation, indicating that more paramagnetic species were generated upon photoexcitation.

### 2.2 Optical Property Investigation

Considering that the photoabsorptivity, optical bandgap, and energy level alignment profoundly impact the photocatalytic efficiency, a set of characterizations were conducted for cross‐validation. The ultraviolet–visible (UV–vis) absorption spectrum of COF‐TPA‐TMTAO exhibited broad absorption with a significant redshift of the absorption onset relative to COF‐TPA‐TMTA and the corresponding small molecule analogs (Figure 3b). The bandgap energies calculated from the Tauc plot are 2.35 and 1.93 eV for COF‐TPA‐TMTA and COF‐TPA‐TMTAO, respectively, indicating that the incorporation of oxygen species is beneficial to narrowing the bandgap (Figure 3c), which was also verified by density functional theory (DFT) calculations. Notably, compared with COF‐TPA‐TMTA, COF‐TPA‐TMTAO possesses more energy levels in each energy band, providing additional opportunities for the ISC process.^[^ ^5a^ ^]^ Mott–Schottky electrochemical measurements were conducted to estimate their energy level alignment. The positive slope indicates n‐type semiconductor behavior for the COFs (Figure 3d,e). The flat band position values of COF‐TPA‐TMTA and COF‐TPA‐TMTAO estimated at 1/*C* ^2^ = 0 (LUMO) are −0.85 and 0.37 V versus NHE, respectively. It is worth noting that the significantly increased LUMO potential of the COF after oxidation can suppress some photoredox processes such as the reduction of O_2_ to O_2_^•−^ (−0.33 eV vs NHE, Figure 3f). DFT calculations of the corresponding COF struts validated the experimentally measured results (Figure S10, Supporting Information). After anchoring the O species on the N ends in triazine, the energy of upper excited states (S*_n_* and T*_n_*) decrease gradually and move closer to the lowest excited states (S_1_ and T_1_), which are conducive to the ISC process (Figures S11 and S12, Supporting Information).

### 2.3 Excitonic Effects Study

**Figure 4:**
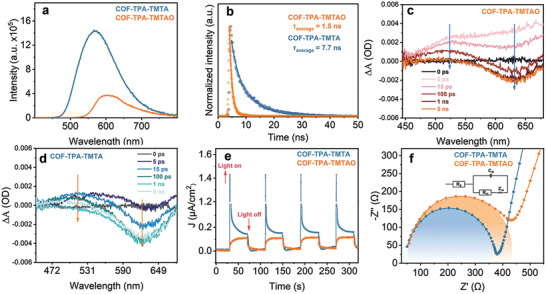
a) Steady‐state PL spectra excited with 375 nm. b) Time‐resolved fluorescence spectra. c,d) TA spectra. e) Photocurrent density versus time recorded under the open‐circuit condition. f) EIS Nyquist plots collected in the dark.

The energy transfer processes that occur under illumination were investigated by recording steady‐state photoluminescence (PL) spectra at an excitation wavelength of 375 nm. After oxidation, the intensity of the PL signal of the sample is significantly lower compared with that of the pristine COF (**Figure** **4**a), suggestive of enhanced competition from the ISC process from singlet to triplet and/or decreased exciton recombination in COF‐TPA‐TMTAO. Time‐resolved fluorescence (TRF) spectra recorded at the corresponding steady‐state emission peaks afforded average radiative lifetimes of 7.7 and 1.5 ns for COF‐TPA‐TMTA and COF‐TPA‐TMTAO, respectively (Figure 4b).^[^ ^19^ ^]^

**Figure 5:**
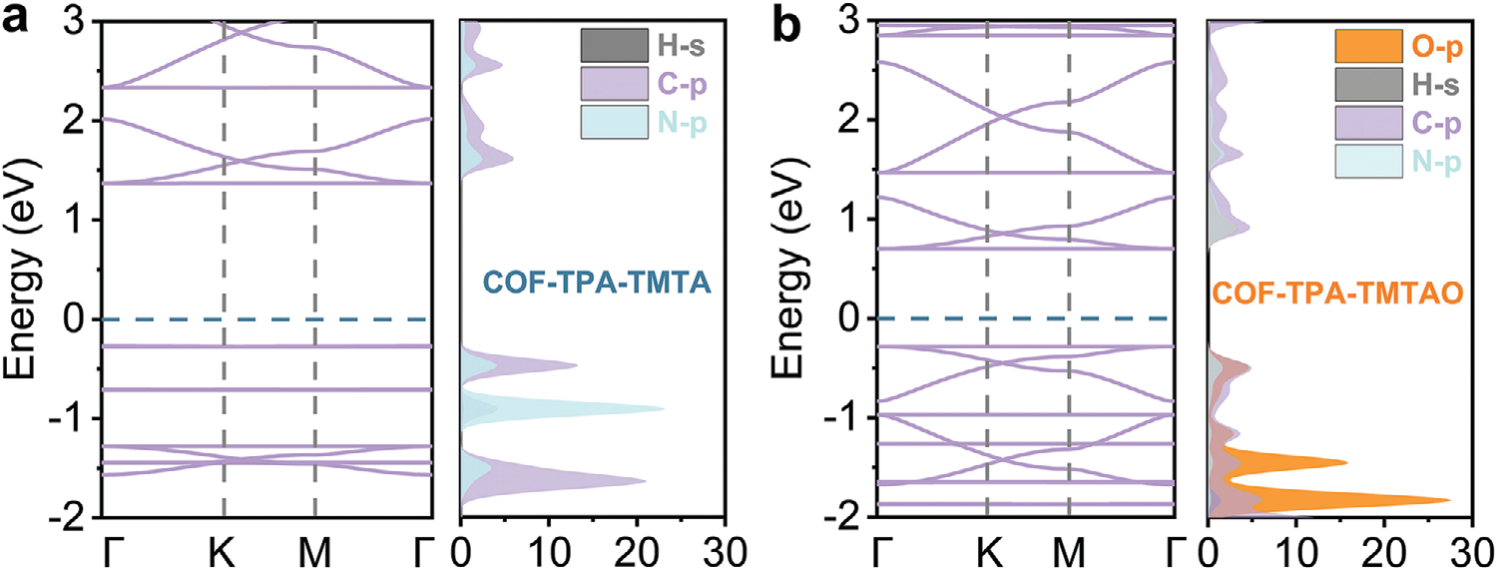
Calculated band structure and partial density of state (PDOS).

To gain insight into the evaluation of the energy transfer dynamics, we performed transient absorption (TA) measurements. Both samples exhibited broad excited‐state absorption at ≈620 nm, showing negative absorbance changes (Figure 4c,d). Two time constants can be resolved, that is, *τ*_1_ = 41.2 ps (54.6%) and *τ*_2_ = 6.3 ps (45.4%) for COF‐TPA‐TMTA, and *τ*_1_ = 9.2 ps (60.5%) and *τ*_2_ = 119.1 ps (39.5%) for COF‐TPA‐TMTAO, respectively, confirming that COF‐TPA‐TMTAO exhibited a longer lifetime of excited state compared to COF‐TPA‐TMTA.^[^ ^20^ ^]^ Considered in combination, the Mott–Schottky plots, PL, and TRF results implied that although electron transfer reactions might play an insignificant role in the photocatalysis of COF‐TPA‐TMTAO, this would not be true for COF‐TPA‐TMTA. The effect of the introduced O atoms was further explored by studying the electronic structures of the COFs with the aid of DFT calculations. The partial density of states (PDOS) profiles revealed that the introduction of O significantly reduces the DOS of the valence band maximum close to the Fermi level, thereby suspending the electron transfer reactions (**Figure** **5**). This is consistent with the characterization mentioned above. These results were further supported by the photocurrent and electrochemical impedance measurements (Figure 4e,f). COF‐TPA‐TMTAO exhibited a weaker photocurrent response and larger charge transfer resistance (i.e., the diameter of the semicircle is wider and the internal resistance is stronger) than that of COF‐TPA‐TMTA.^[^ ^19^ ^]^

In an attempt to gain additional insight into the distinct excitonic effects in the COFs before and after oxidation, their molecular oxygen activation performance was evaluated. The activation of O_2_ to yield various reactive oxygen species (ROS), including singlet oxygen (^1^O_2_), the superoxide anion radical (O_2_^•−^), and the hydroxyl radical (•OH) has been proven.^[^ ^4b^ ^]^ Generally, the energy transfer process accounts for the formation of ^1^O_2_, during which singlet excitons transform to triplets and then react with O_2_. Electron transfer is responsible for the generation of O_2_^•−^ and •OH. According to the above spectroscopic studies and DFT calculations, COF‐TPA‐TMTAO is expected to exhibit superior ^1^O_2_ generation efficiency, but O_2_^•−^ and •OH production yields that are inferior to those of COF‐TPA‐TMTA. To identify the type of generated ROS species, specific indicators were used. Only ^1^O_2_ species were identified for COF‐TPA‐TMTAO, whereas both ^1^O_2_ and O_2_^•−^ species were detected by COF‐TPA‐TMTA (Figures S13–S15, Supporting Information). To quantify the generation efficiency of ^1^O_2_, quantum yields of ^1^O_2_ for the COF materials were calculated, which revealed that the efficiency of COF‐TPA‐TMTAO was more than five times greater than that of COF‐TPA‐TMTA (Figure S16, Supporting Information). These results were further validated by comparing their performance in terms of the oxidation of *α*‐terpinene to ascaridole, as the contribution of various ROS to the reaction can be identified from the selectivity of various products.^[^ ^4b^ ^]^ The results in **Table** **1** indicate that the activity and selectivity of COF‐TPA‐TMTAO toward *α*‐terpinene were very high (99% and 90%, respectively), supporting the critical role of ^1^O_2_. In sharp contrast, COF‐TPA‐TMTA only afforded *α*‐terpinene conversion of 22% with ^1^O_2_‐associated ascaridole selectivity of 60%, alluding to the different photocatalytic transformation paths of ROS generation.

**Table 1:** Catalytic Evaluation of *α*‐terpinene into ascaridole over COF‐TPA‐TMTA and COF‐TPA‐TMTAO

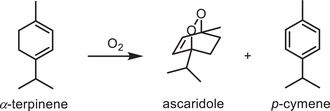				
Entry^a)^	Catalyst	Conversion [%]^b)^	Selectivity [%]^b)^	
			Ascaridole	*p*‐Cymene
1	COF‐TPA‐TMTA	22	60	40
2	COF‐TPA‐TMTAO	99	90	10

^a)^ Standard reactions were conducted with *α*‐terpinene (0.1 mmol) and COF material (5 mg) in DMF (4 mL) at room temperature under blue LED irradiation (40 W) for 60 min

^b)^ Conversion and selectivity were determined by GC analysis.

### 2.4 Catalytic Evaluation

Given the abovementioned results, the photooxidation of sulfides was chosen for confirming the enhanced ISC process based on the following considerations:^[^ ^21^ ^]^ 1) These oxidation reactions are important, considering the central role of sulfoxides in pharmacy and living organisms. 2) The reaction involves ^1^O_2_ when the reactions are carried out in a protic solvent such as methanol. Photocatalytic thioanisole oxidation to methyl phenyl sulfoxide was conducted under blue LED irradiation. Control experiments revealed that the supply of photocatalysts and light is crucial, as no product was detected in the absence of either photocatalyst or light (**Table** **2**, entries 1 and 2). In stark contrast to the low oxidized product yield of COF‐TPA‐TMTA (15%, Table 2, entry 3), COF‐TPA‐TMTAO exhibited an exponential enhancement in the methyl phenyl sulfoxide yield >99% (Table 2, entry 4), which was more than six times higher than that of the pristine COF. Prolonging the reaction time to 24 h, only 55% of thioanisole was converted by COF‐TPA‐TMTA (Figure S17, Supporting Information). A great enhancement of catalytic efficiency after oxidation was also observed in their molecular counterparts. TMTACN and TMTACNO afforded the methyl phenyl sulfoxide yields of 0.5% and 45%, respectively, under otherwise identical conditions (Table 2, entries 5 and 6). Nonetheless, both of them show lower activities in relation to the corresponding COF analogies, underscoring the role of extending the conjugation of photosensitizers. Strikingly, the physical mixture of COF‐TPA‐TMTA and (NH_4_)_2_S_2_O_8_ afforded a methyl phenyl sulfoxide yield of only 49% (Table 2, entry 7). The results suggest the formation of inner molecule donor–acceptor pairs, which accelerated the ISC process and thus achieved an excellent efficiency of triplets. To gain insight into the reaction pathway, the role of various intermediates in this light‐driven oxidation was investigated. A significantly decreased yield (4%, Table 2, entry 8) was observed with triethylenediamine (DABCO) as a ^1^O_2_ scavenger.^[^ ^4^ ^]^ Using phenothiazine (PHT) as an exciton scavenger which can quench the excitonic processes, negligible conversion was detected.^[^ ^22^ ^]^ When ethylenediaminetetraacetic acid disodium (Na_2_EDTA) as a hole scavenger and isopropanol (*i*‐PrOH) as an •OH scavenger was added to the reaction mixture, only a slight decrease in the conversion of thioanisole was observed, suggesting the minor roles of these species (Table 2, entries 9–11). To confirm the role of ^1^O_2_ in the reaction, a trapping agent, 2,2,6,6‐tetramethyl‐1‐piperidine (TEMP), was introduced to detect the generated ^1^O_2_ (Figure S18, Supporting Information). The EPR spectrum displays a quantitative resemblance to the three‐line spectrum of the trapped ^1^O_2_ in TEMP, validating the participation of ^1^O_2_ in the catalytic cycle. In contrast, all these ROS scavengers can depress the reaction, suggesting that ^1^O_2_, •OH, and O_2_^•−^ are involved in the COF‐TPA‐TMTA catalyzed transformations. Moreover, spin trapping studies confirmed the formation of both ^1^O_2_ and O_2_^•−^ under illumination (Table S3 and Figure S19, Supporting Information). These results collectively indicated that the creation of long‐pair donor–acceptor bonds is a feasible way to steer the evolution of highly reactive intermediates toward the desired pathways.

**Table 2:** Catalytic evaluation of oxidation of thioanisole into methyl phenyl sulfoxide over various conditions

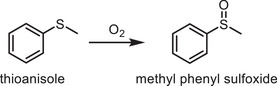				
Entry^a)^	Catalyst	Additive	Light	Yield [%]^b)^
1	–	−	+	n.d.
2	COF‐TPA‐TMTAO	−	−	n.d.
3	COF‐TPA‐TMTA	−	+	15
4	COF‐TPA‐TMTAO	−	+	99
5	TMTACN	−	+	0.5
6	TMTACNO	−	+	45
7	COF‐TPA‐TMTA	(NH_4_)_2_S_2_O_8_	+	49
8	COF‐TPA‐TMTAO	DABCO	+	4
9	COF‐TPA‐TMTAO	PHT	+	0
10	COF‐TPA‐TMTAO	Na_2_EDTA	+	82
11	COF‐TPA‐TMTAO	*i*‐PrOH	+	93
12	COF‐TPA‐TMTAO	−	Sunlight	92

^a)^ Standard reactions were conducted with thioanisole (0.5 mmol), COF material (5 mg), and additive (2.5 mmol) in MeOH (4 mL) at room temperature under blue LED irradiation (40 W) for 30 min

^b)^ Yields were determined by ^1^H NMR analysis. n.d. = not detected.

To expand the applicability of COF‐TPA‐TMTAO, experiments were performed under direct sunlight. A 92% sulfoxide product yield was achieved within 120 min, implying great promise for low‐environmental‐impact transformations (Table 2, entry 12). Further recycling experiments for COF‐TPA‐TMTAO suggest the well‐retained activity for five catalytic runs (Figure S20, Supporting Information). Advantageously, the material is stable during catalysis, as supported by the retained XRD pattern (Figure S21, Supporting Information).

With a preferred set of conditions in hand, we sought to evaluate the scope of the sulfide components. Six additional thioanisole derivatives with various electron‐donating groups (—Me and —OMe) and electron‐withdrawing groups (—F, —Cl, —Br, and —NO_2_) at the *para*‐position were investigated. The electron‐rich thioanisole derivatives exhibited faster reaction rates than electron‐poor ones. Further expansion of the substrate scope demonstrated a broad substrate tolerance. Under standard conditions, tetrahydrothiophene, *tert*‐butyl sulfide, and benzyl sulfide can be fully converted to the corresponding sulfoxide. Notably, the protocol allowed for the oxidation of 2‐chloroethyl ethyl sulfide, which is the surrogate for nerve agents (Table S4, Supporting Information).

## 3 Conclusion

In summary, we have demonstrated that the installation of O species on the N ends of the olefin‐linked triazine COF has a profound effect on the material's photoabsorptivity, band structure, and frontier orbital, thereby showcasing a practical way to control the evolution of highly reactive intermediates synthetically. The built‐in lone‐pair donor–acceptor bonds offer a narrow bandgap to harvest visible light, promote exciton migration, and enhance the triplet production efficiency to boost the ^1^O_2_ production efficiency and subsequent oxidation transformation. Additionally, this strategy can be readily extended to other triazine contained materials and significantly enhanced photocatalytic activity after creating lone‐pair donor–acceptor bonds was observed (Figure S22, Supporting Information). Our results have thus established a new protocol for designing efficient photocatalysts, especially those containing triazine moieties. Beyond practicability and sustainability, the development of strategy would inspire further advancement of efficient artificial photosynthesis.

## Conflict of Interest

The authors declare no conflict of interest.

## Supporting information

Supporting Information

## Data Availability

The data that supports the findings of this study are available in the Supporting Information of this article.
