# Supplementary material for: Turn‐On Photocatalysis: Creating Lone‐Pair Donor–Acceptor Bonds in Organic Photosensitizer to Enhance Intersystem Crossing
Source: Adv Sci (Weinh). 2021 Aug 2;8(18):2100631. doi: 10.1002/advs.202100631 (PMC8456219; doi:10.1002/advs.202100631)
Supplement: Supplementary file 1 — Supporting Information [file ADVS-8-2100631-s001.pdf]

© 2021 Wiley-VCH GmbH

ADVANCED SCIENCE
Open Access

Supporting Information

for *Adv. Sci.,* DOI: 10.1002/advs.202100631

Turn-on Photocatalysis: Creating Lone-Pair Donor-Acceptor Bonds in Organic Photosensitizer to Enhance Intersystem Crossing

Mingjie Liu, Junnan Liu, Kai Zhou, Jingwen Chen,Qi Sun, Zongbi Bao, Qiwei Yang, Yiwen Yang, Qilong Ren, Zhiguo Zhang**

((Supporting Information can be included here using this template))

Supporting Information

Turn-on Photocatalysis: Creating Lone-Pair Donor-Acceptor Bonds in Organic Photosensitizer to Enhance Intersystem Crossing

Mingjie Liu, Junnan Liu, Kai Zhou, Jingwen Chen,Qi Sun,*Zongbi Bao, Qiwei Yang, Yiwen Yang, Qilong Ren, Zhiguo Zhang*

Chemicals

Commercially available reagents and solvents were purchased in high purity and used without further purification. 2,4,6-Trimethyl-1,3,5-triazine (TMTA) and terephthalaldehyde were purchased from Jilin Chinese Academy of Sciences-Yanshen Technology Co., Ltd..

General methods

Powder X-ray diffraction (PXRD) data were collected on a Bruker AXS D8 Advance A25 Powder X-ray diffractometer (40 kV, 40 mA) using Cu Kα (λ= 1.5406 Å) radiation. Scanning electron microscopy (SEM) images were collected using a Hitachi SU 8010. IR spectra were recorded on a Nicolet Impact 410 FTIR spectrometer. Thermogravimetric analysis (TGA) analyses were performed on a TA instrument SDT 650 under nitrogen atmosphere at a heating rate of 10 °C min^{-1} over a temperature range of 40-800 °C. N_2 sorption measurements were carried out on a TriStar II Plus instrument at 77 K. Samples were degassed at 120 °C for 24 h before tests.^{1}H NMR spectra were recorded on a Bruker Avance-400 (400 MHz) spectrometer. Chemical shifts are expressed in ppm downfield from TMS at δ=0 ppm, and *J* values are given in Hz.^{13}C (100.5 MHz) cross-polarization magic-angle spinning (CP-MAS) was recorded on a Varian infinity plus 400 spectrometer equipped with a magic-angle spin probe in a 4-mm ZrO_2 rotor. Matrix-assisted laser desorption/ionization time-of-flight mass spectrometry (MALDI-TOF MS) was recorded on a Bruker MALDI-TOF using trans-2-[3-(4-tert-Butylphenyl)-2-methyl-2-propenylidene] malononitrile as a substrate. UV-Vis diffuse reflectance spectra were recorded on a SHIMADZU UV-2600 spectrometer. Liquid UV-Vis spectra were performed on a SHIMADZU UV-2600.The fluorescence spectra were measured under 380 nm excitation in the 380-600 nm in 2 nm steps using a LifeSpec-II fluorescence spectrometer.The steady-state fluorescence and lifetime measurements were carried out on an Edinburgh FLS1000. Time-correlated single photon counting (TCSPC) measurements were performed on a LifeSpec-II fluorescence spectrometer employing an EPL485 pulsed laser diode and a Hamamatsu H5773-04 photomultiplier.

Material synthesis

Synthesis of 2,4,6-tris[(1E)-2-(4- cyanophenyl)ethenyl]-1,3,5-triazine [TMTACN]

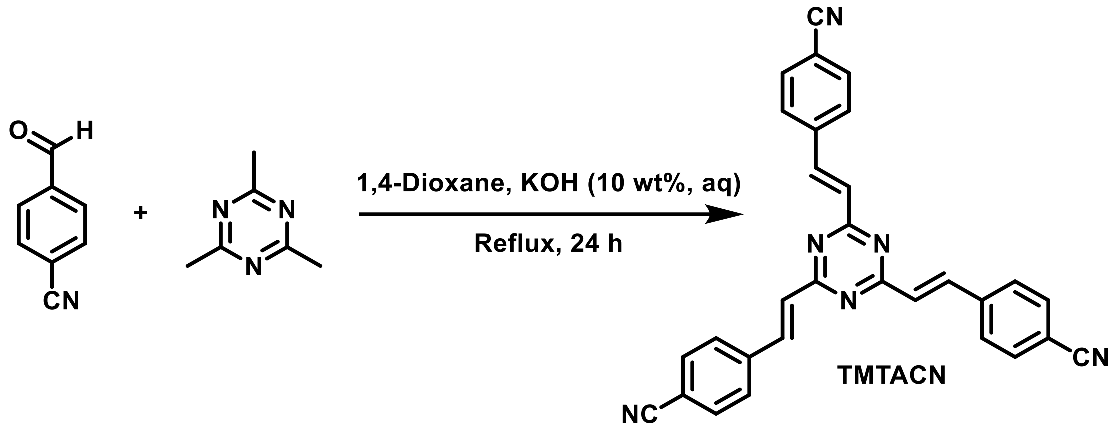

2,4,6-tris[(E)-4-cyanostyryl]-1,3,5-triazine [TMTACN]

4-Cyanobenzaldehyde (524.5 mg, 4 mmol), 2,4,6-trimethyl-triazine (123.2 mg, 1 mmol), and KOH aqueous solution (2 mL, 10 wt%) were dissolved in 10 mL of 1,4-dioxane. The resulting mixture was heated to reflux for 24 h, yielding a white solid. The mixture was cooled to room temperature, filtered, and washed with water, methanol, and dichloromethane in sequence. The resulting white solid was dried under vacuum to afford **TMTACN** (361 mg, yield 78%).^{1}H NMR (400 MHz, $CDCl_3$, 298 K, TMS): δ 8.28 (d, *J* = 15.9 Hz, 3H), 7.76 (q, *J* = 8.5 Hz, 12H), 7.26 (d, *J* = 16.0 Hz, 3H). MALDI-TOF-MS: *m/z* calculated for $C_{30}H_{19}N_6$ [M$^+$]: 463.16, found 463.15.

Synthesis of 2,4,6-tris((E)-4-cyanostyryl)-1,3,5-triazine 1,3,5-trioxide [TMTACNO]

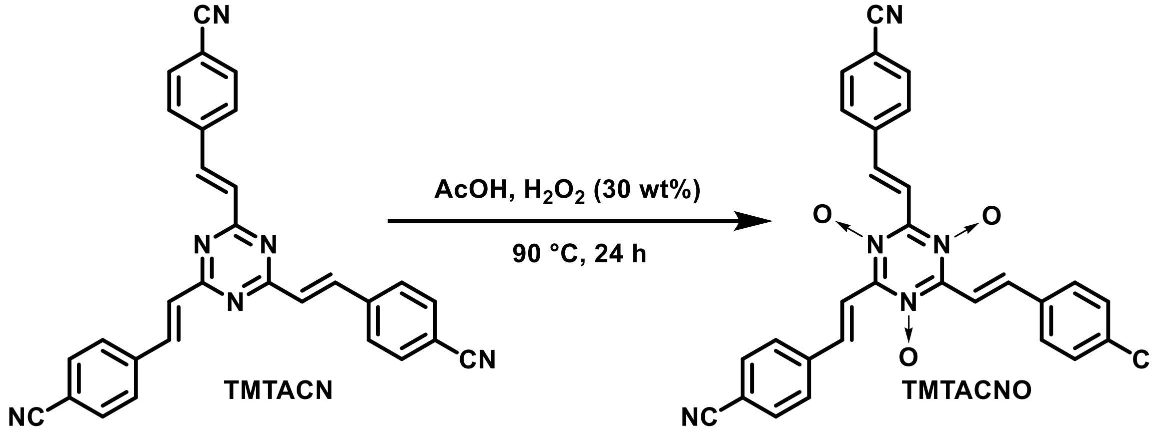

2,4,6-tris((E)-4-cyanostyryl)-1,3,5-triazine 1,3,5-trioxide [TMTACNO]:

2,4,6-Tris[(E)- 4-cyanostyryl]-1,3,5-triazine (200 mg) and H_2O_2 (30 wt%, 4 mL) were suspended in AcOH (10 mL). The resulting mixture was stirred at 90 °C for 24 h, which was poured into cold water and filtered. The orange filter cake was collected and redissolved in THF, dried over Na_2SO_4,and evaporated under reduced pressure, giving the crude compound which was purified by flash chromatography with dichloromethane/tetrahydrofuran (v/v: 100/1) as eluent to afford the **TMTACNO**as an orange solid (110 mg, yield 50%). ^{1}H NMR (400 MHz, $CDCl_3$, 298 K, TMS): δ 8.21 (d, *J* = 16.3 Hz, 3H), 7.68 (s, 12H), 7.47 (s, 3H), MALDI-TOF -MS: *m/z* calculated for $C_{30}H_{19}N_6O_3$ [M+H]$^+$: 512.16, found 512.66.

Synthesis of COF-TPA-TMTA

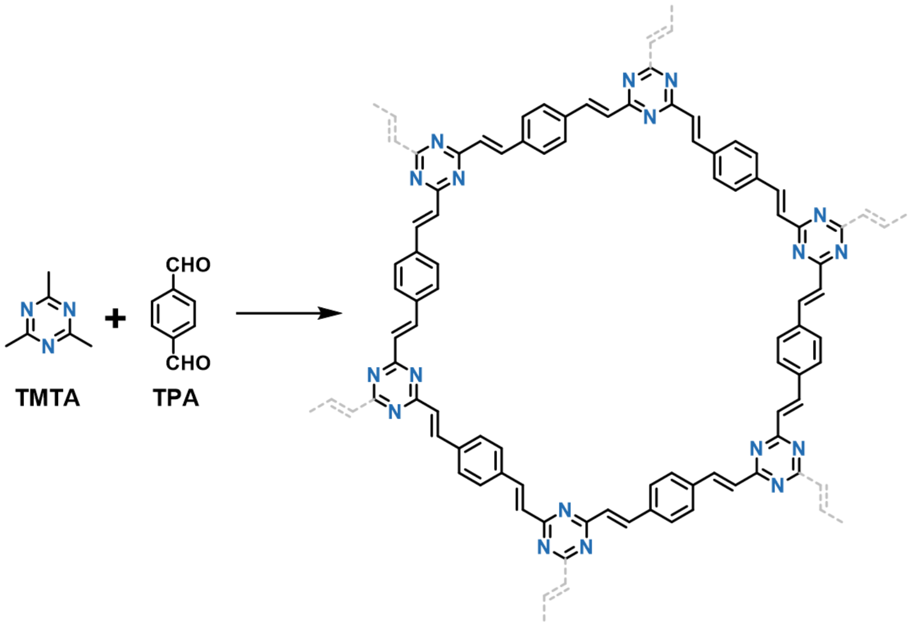

A Schlenk tube (350 mL) was charged with **TMTA** (554.2 mg, 4.5 mmol), **TPA** (905.4 mg, 6.75mmol), and KOH (757.4 mg, 13.5 mg) in 90 mL of a 3/7 v/v solution of 1,2-dichlorobenzene /*n*-butyl alcohol. After the solid was completely dissolved, the reaction mixture was heated at 120 °C for 3 days to afford a yellow precipitate, which was isolated by filtration and washed with methanol using Soxhlet extraction for 24h. The product was dried under vacuum at 120 ºC for 12 h. The titled COF-TPA-TMTA sample was achieved as a pale-yellow powder in a yield of approximately 80% (Table S1).

Synthesis of COF-TPA-TMTAO

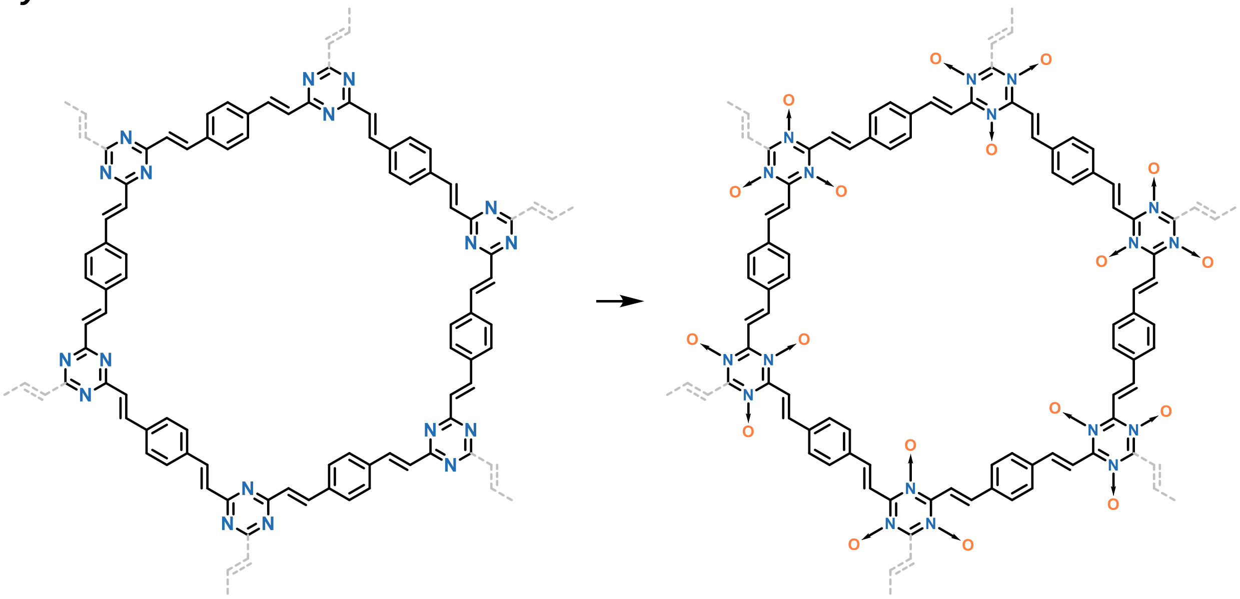

COF-TPA-TMTA (100 mg) was suspended in water (10 mL), and the mixture was heated to 50 ºC. A solution of $(NH_4)_2S_2O_8$ (10 wt%) (10 mL) was added to the suspension dropwise and heated at 50 ºC for 24 h.As the reaction proceeded, the initial yellowish reaction mixture gradually changed to orange. The resulting precipitate was isolated by filtration, washed with methanol, and dried under vacuum overnight to give an orange powder in approximately 85% yield (Table S2).

Catalytic evaluation

General Procedure for Photocatalytic Oxidation of Sulfides.
A Schlenk tube (10 mL) was charged with MeOH (4 mL), sulfides (0.5 mmol), and photocatalyst (5 mg). The mixture was saturated with O_2 and magnetically stirred at room temperature under irradiation of 460 nm LED lamp (40 W). Once the reaction was completed, the photocatalyst was collected by filtration, and the solvent was removed under vacuum. The conversions and selectivity were determined by ^{1}H NMR based on the ratio between integrated peaks of products and substrate.

DFT Calculations

HOMO-LUMO distributions of the COF struts were calculated by density functional theory (DFT) with B3LYP/6-31 G(d) basis. The Natural Transition Orbital (NTO) particle-hole representation of the repeat units of the COF materials (COF-TPA-TMTA and COF-TPA-TMTAO) and distribution of excited states (T_n and S_n) were optimized by time-dependent density functional theory (TD-DFT) with B3LYP/6-31 G(d) basis set implemented in Gaussian 09 program package [M. J. Frisch, G. W. Trucks, H. B. Schlegel, G. E. Robb, J. R. Cheeseman, G. Scalmani, V. Barone, B. Mennucci, G. A. Petersson, et al., Gaussian 09, revision A. 01, Gaussian, Inc.: Wallingford, CT, 2009]. The calculated data was further analyzed in Multiwfn [T. Liu, J. Comput. Chem., 2012, 33, 580-592 (2012)] [Z. Liu, Carbon, 2020, 165, 461-467].The band-structure and density of state (DOS) calculations of the COFs were carried out using the projected augmented wave (PAW) method [P. E. Blöchl, Phys. Rev. B, 1994, 50, 17953-17979.], as implemented in the Vienna ab-initio Simulation Package (VASP) [G. Kresse, J. Furthmüller, Phys. Rev. B, 1996, 54, 11169-11186.] [G. Kresse, J. Hafner, Phys. Rev. B, 1994, 49, 14251-14269.]. The exchange-correlation interaction [J. P. Perdew, K. Burke, M. Ernzerhof, Phys. Rev. Lett., 1996, *77*, 3865-3868.] is treated with the generalized gradient approximation (GGA) of the Perdew-Burke-Ernzerhof (PBE) scheme. The cut-off energy was set to 450 eV for the plane-wave expansion of wavefunctions, and the convergence threshold was set to 10^{-4} and 0.02 eV/Å for energy and force, respectively. The Brillouin zone was sampled by a Γ centered 3×3×1 Monkhorst-Pack (MP) grid for structural optimizations, and a denser 6×6×1 MP grid was used for electronic property calculations.

Electrochemical measurements

All electrochemical measurements were performed with a CHI660E workstation (ShangHai ChenHua, China) using a standard three-electrode system. The working electrode was prepared as follows: the COF material (2 mg) was dispersed in a mixed solution of *n*-butyl alcohol (1 mL) and polytetrafluoroethylene (PTFE) (60 wt%, 20 μL). The resulting mixture was ultra-sonicated for 30 min to get a homogeneous slurry. Subsequently, 50 μL of the slurry was coated onto a glassy carbon electrode (3 mm) and dried at 50 °C. For Mott-Schottky tests, the Ag/AgCl electrode was employed as the reference electrode, and a platinum was used as the counter electrode, respectively. Theperturbation was 5 mV with a frequency of 1500, 2000, and 2500 Hz. The electrochemical impedance measurements were carried out under the open-circuit condition with an alternating current amplitude of 5 mV in the frequencies range of 0.01 Hz to 10^5 Hz. The photocurrent experiments were executed under visible light generated by a 300 W Xenon lamp and chopped manually.Cyclic voltammetry measurement: The working electrode was prepared by the same procedure mentioned above. Tetrabutylammonium hexafluorophosphate

(0.1 M, acetonitrile) was used as an electrolyte. The counter and reference electrodes were Pt wire and Ag/$AgNO_3$. Ferrocene was used as a standard to calculate the energy levels vs. vacuum.

Electron paramagnetic resonance (EPR) Characterization:

Powder samples:2 mg of COFs were separately added to nuclear magnetic tubes (5 mm o.d.) with caps. The sealed tube was then mounted in a Varian E-109 spectrometer fitted with a cavity resonator. The continuous wave (CW) EPR spectrum was acquired with an observe power of 12.5 mW and modulation amplitude of 2 G. The main frequency is 9.550 GHz. The scan range is 200 G, from 3400 G to 3600 G. Signal average time is about 5 min. The sealed tube was then irradiated with a 300 W xenon for 30 min, followed by the same EPR studies.

Spin trapping studies for $O_2^{\bullet-}$ detection: 2 mg of COFs were suspended in 2 mL of methanol and irradiated for 30 min, followed by the addition of 20 μmol of DMPO. The resulting mixture was loaded in a borosilicate capillary tube, followed by mounting in a Varian E-109 spectrometer fitted with a cavity resonator. The continuous wave (CW) EPR spectrum was acquired with an observe power of 12.5 mW and modulation amplitude of 2 G. The main frequency is 9.550 GHz. The scan range is 100 G, from 3300 G to 3400 G. Signal average time is around 5 min.

Spin trapping studies for $^1O_2$ detection:The experiments were performed followed the same procedures as for $O_2^{\bullet-}$ detection except that 50 μM of TEMP was used instead of 50 μM DMPO.

Transient Absorption spectra measurement (TA):

For transient absorption (TA) measurements, the COFs were pressed into transparent tablet together with KBr in a mass ratio of 1: 300 (COFs : KBr), and then experiments were performed on a femtosecond pump-probe setup consisting of Helios (Ultrafast Systems LLC) spectrometers and a regeneratively amplified Ti : sapphire laser source (Coherent Legend, 800 nm, 150 fs, 5 mJ per pulse, and 1 kHz repetition rate) in the transmission mode. A pump light of 350 nm was generated by this laser source, and the power intensity can be adjusted by a range of neutral-density filters, which were fixed at 125 μJ cm^{-2} in these measurements. The delay time window between the pump and probe pulse from 0 to 7 ns was controlled by a motorized delay time stage.

Steady-state/time-resolved fluorescence spectra measurement:

The steady-state fluorescence spectra were performed on the FLS1000 steady/transient fluorescence spectrometer. The COFs powders were irradiated at 375 nm using a xenon lamp source, during which the wavelength before 400 nm was cut off by a filter. The time-resolved fluorescence measurements were carried out using a time-correlated single-photon counting system, the signal was recorded using an Edinburgh FLS 920 spectrometer, and COFs were excited with a picosecond diode laser (Edinburgh Instruments EPLED-375 375 nm, 10 MHz,140 Mw) with a pulse width of 75 ps.

Calculation of the quantum yield of singlet oxygen:

1,3-diphenyl-isobenzofuran (DPBF) was used as the $^1O_2$ trapping agent. 3 mg of the COF materials were introduced to 20 mL of0.1 mM DPBF air saturated MeOH solution.The resulting system was irritatedunder a blue LED light (40 W). The fluorescence signal at 450 nm (excited 380 nm) was recorded at various irradiation times to obtain the decay rate of the photosensitizing process.

The$^1O_2$ quantum yield of COFs in MeOH (Φ_{COFs}) was calculated using the following formulas:

$$\frac{-\Delta[\mathbf{DPBF}]}{t} = \frac{I_0 - I_t}{t} = I_{in}\Phi_{ab}\Phi_{\Delta}\Phi_r$$

$$\frac{k}{k^s} = \frac{\Phi_{ab}}{\Phi_{ab}^s} = \frac{\Phi_{\Delta}}{\Phi_{\Delta}^s}$$

where I_{in} is the incident monochromatic light intensity, Φ_{ab} is the light-absorbing efficiency, Φr is the reaction quantum yield of $^1O_2$ with DPBF, t is the reaction time, I_0 and I_t are the fluorescence intensities of DPBF before and after the addition of COFs, k is the slope, and superscript s stands for the slop of $[Ru(bpy)_3]Cl_2{\cdot}6H_2O$ (Φ = 0.81 in MeOH).

Identification of ROS species:

•*OH:* The stock solution of coumarin-3-carboxylic acid (0.1 mM) was prepared by using air-saturated MeOH. 3 mg of COF materials were introduced to a10 mL of the coumarin-3-carboxylic acid solution. The suspensions were continuously stirred in dark for 30 min before being irradiated under a blue LED light (460 nm, 40 W). After irradiation for 30 min, the COFs were separated by filtration and the resulting solution was determined by UV-Vis to check the characteristic peak associated with the adduct of •OH and indicator.

$O_2^{\bullet-}$: 3 mg of COF materials were introduced into air saturated 10 mL of 0.1 mM nitrotetrazolium blue chloride MeOH solution. The suspensions were continuously stirred in dark for 30 min before being irradiated under a blue LED light (460 nm, 40 W). After irradiation for 30 min, the COFs were separated by filtration and the resulting solution was determined by UV-Vis to check the characteristic peak associated with the adduct of $O_2^{\bullet-}$ and indicator.

*$^1O_2$:*3 mg of COF materials were introduced into air saturated 10 mL of 0.1 mM singlet oxygen sensor green MeOH solution. The suspensions were continuously stirred in dark for 30 min before being irradiated under a blue LED light (460 nm, 40 W). After irradiation for 30 min, the COFs were separated by filtration and the resulting solution was determined by the fluorescence spectroscopy to check the characteristic peak associated with the adduct of $^1O_2$ and indicator.

Table S1. Fractional atomic coordinates for the eclipsed (AA) stacking unit cell of COF-TPA-TMTA.
Crystal system: Hexagonal, Space group: *P6/m*,International tables number: 175,$a = b = 22.8204$ Å, $c = 3.4472$ Å,$\alpha = \beta = 90^o$, $\gamma = 120^o$

**Atom**	***x/a***	***y/b***	***z/c***
H1	0.17890	0.51924	0.5
H2	0.16196	0.64401	0.5
H3	0.04882	0.62437	0.5
H4	0.92741	0.55210	0.5
C1	0.14549	0.59048	0.5
C2	0.19274	0.57188	0.5
C3	0.07124	0.54280	0.5
C4	0.04216	0.47192	0.5
C5	0.26570	0.62158	0.5
C6	0.97192	0.42994	0.5
N1	0.31083	0.59935	0.5

Table S2. Fractional atomic coordinates for the eclipsed (AA) stacking unit cell of COF-TPA-TMTAO.
Crystal system: Hexagonal, Space group: *P6/m*,International tables number: 175,$a = b = 23.2883$ Å, $c = 3.4250$ Å,$\alpha = \beta = 90^o$, $\gamma = 120^o$

**Atom**	***x/a***	***y/b***	***z/c***
H1	0.17573	0.52824	0.5
H2	0.15218	0.64508	0.5
H3	0.04203	0.62088	0.5
H4	0.92450	0.54597	0.5
C1	0.14180	0.59481	0.5
C2	0.19084	0.57996	0.5
C3	0.06952	0.54478	0.5
C4	0.04401	0.47567	0.5
C5	0.26526	0.62682	0.5
C6	0.97564	0.43188	0.5
N1	0.30540	0.59936	0.5
O1	0.46487	0.74265	0.5

Table S3. Photocatalytic Oxidation of Various Sulfides over COF-TPA-TMTA.[a]

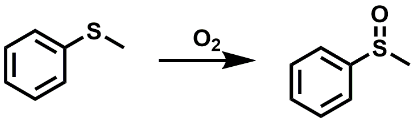

entry	catalyst	scavenger	light	yield (%)[b]
1	COF-TPA-TMTA	-	+	44
2	COF-TPA-TMTA	Na_2EDTA	+	20
3	COF-TPA-TMTA	Phenothiazine	+	2
4	COF-TPA-TMTA	Isopropanol	+	25
5	COF-TPA-TMTA	Triethylenediamine	+	n.d.

[a]General reaction conditions: COF-TPA-TMTA (5 mg), sulfide compound (0.5 mmol), sacverger (2.5 mmol) methanol (4 mL), LED (40 W, 460 nm), O_2, and RT. [b]The selectivity was calculated based on the ratio of methyl phenyl sulfoxide and methyl phenyl sulfoxide in the products, which were determined by ^1H NMR.

Table S4. Photocatalytic Oxidation of Various Sulfides over COF-TPA-TMTA.[a]

entry	substrate	product	time (min)	yeld (%)[b]
1	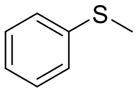	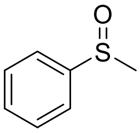	30	99
2	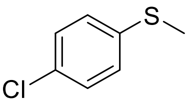	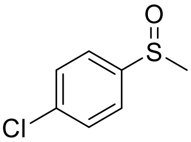	90	98
3	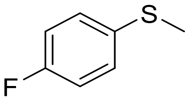	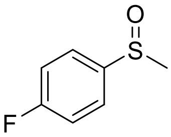	90	99
4	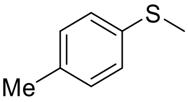	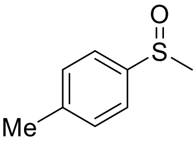	60	99
5	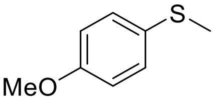	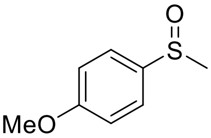	60	99
6	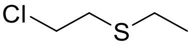	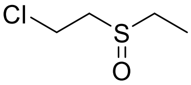	60	99
7	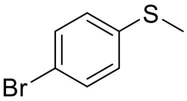	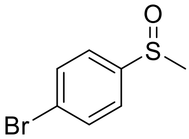	90	93
8	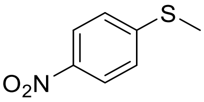	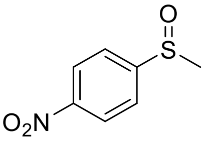	120	82
9	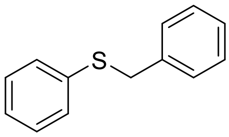	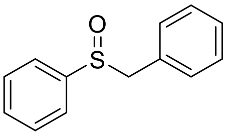	120	97
10	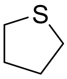	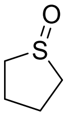	60	99
11	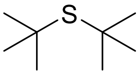	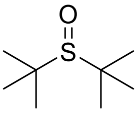	60	99
12	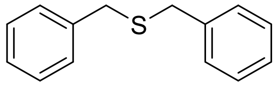	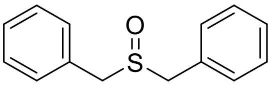	90	98%

[a]General reaction conditions: COF-TPA-TMTAO (5 mg), sulfide compound (0.5 mmol), methanol (4 mL), LED (40 W, 460 nm), O_2, and RT.[b]The selectivity was calculated based on the ratio of methyl phenyl sulfoxide and methyl phenyl sulfoxide in the products, which were determined by ^1H NMR.

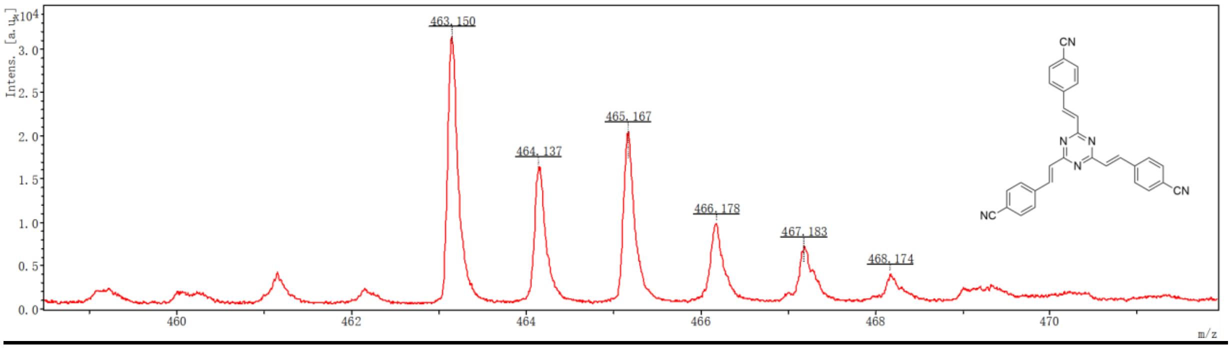

Figure S1. MALDI-TOF mass spectrum of TMTACN.

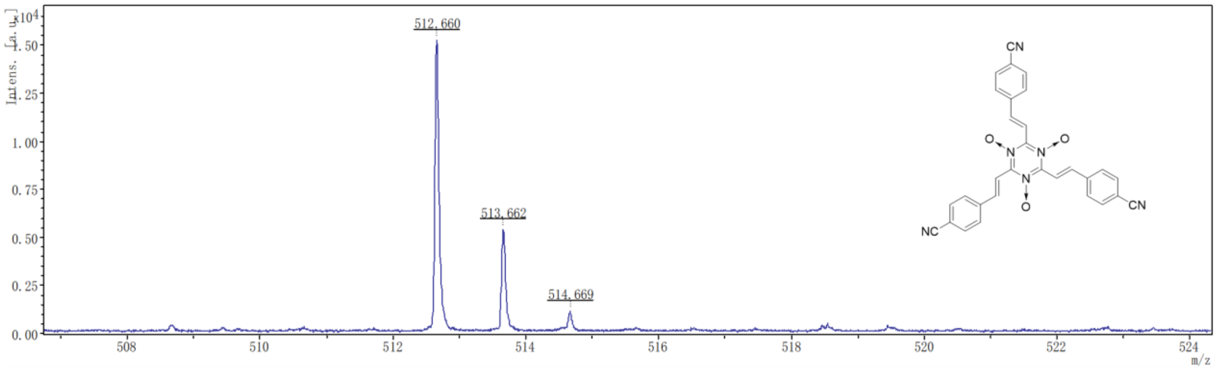

Figure S2. MALDI-TOF mass spectrum of TMTACNO.

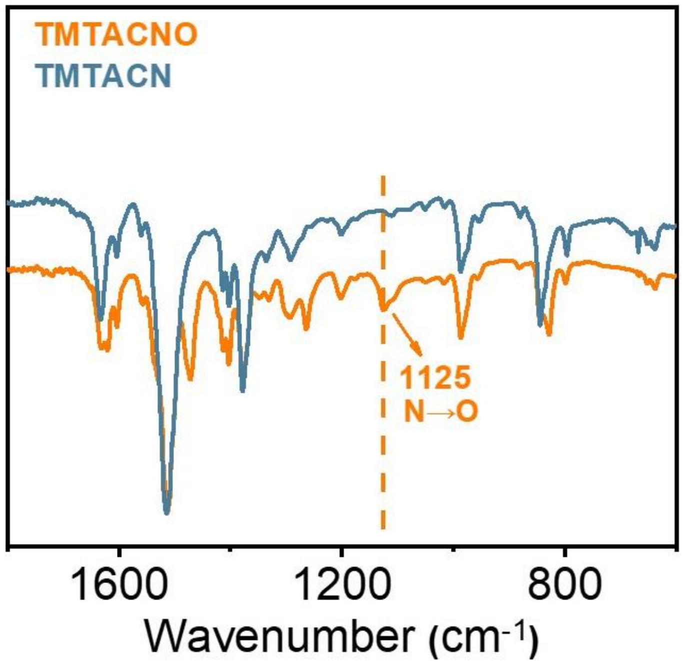

Figure S3. FT-IR spectra of TMTACN and TMTACNO.

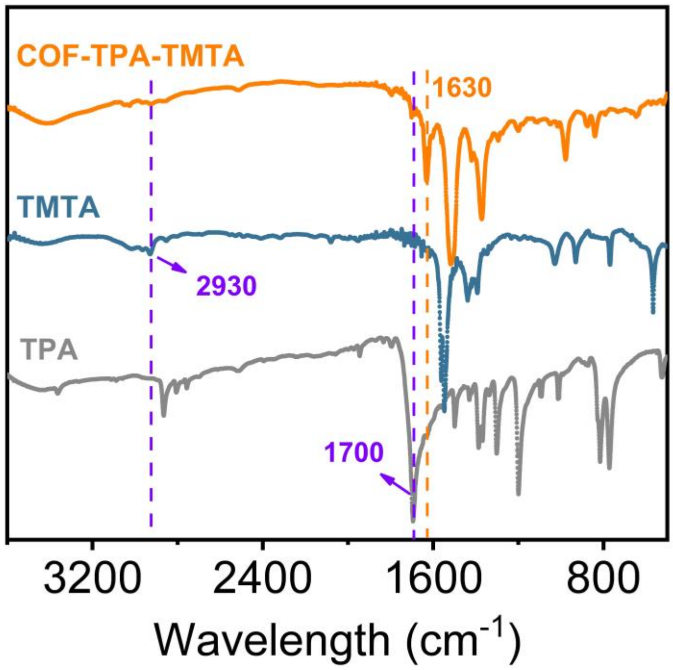

Figure S4. FT-IR spectra of monomers and COF-TPA-TMTA.

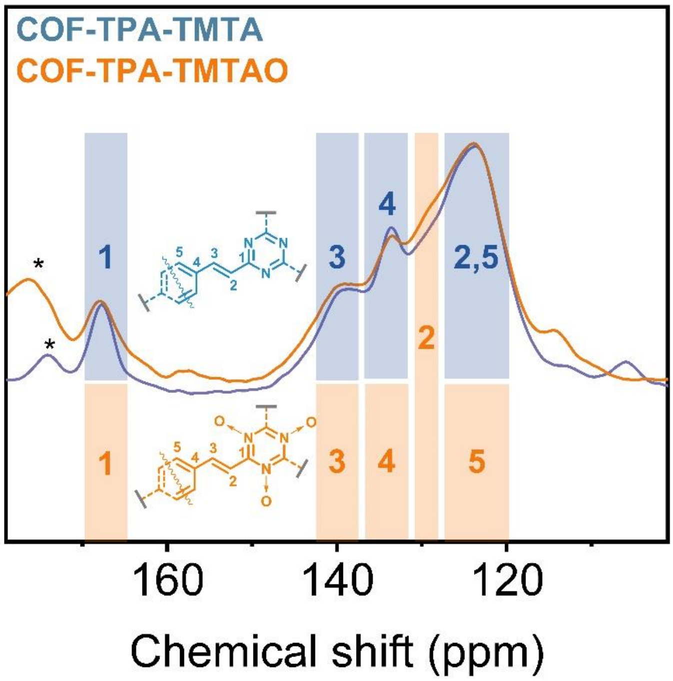

Figure S5. Solid-state ^{13}C NMR spectra of COF-TPA-TMTA and COF-TPA-TMTAO.

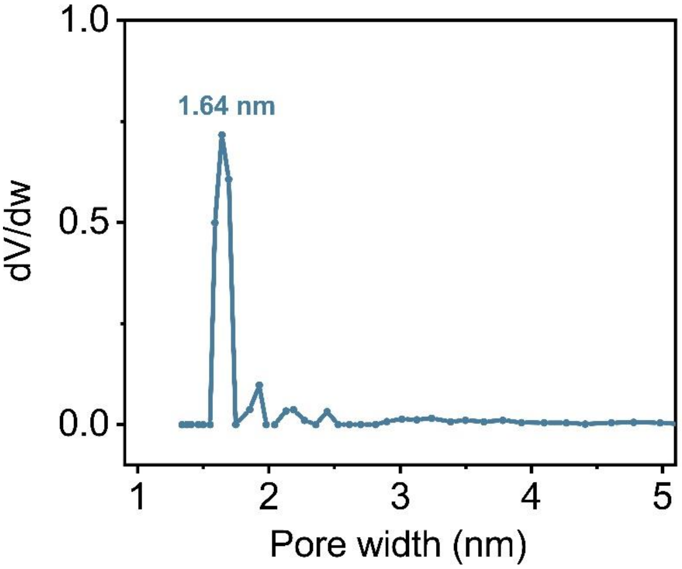

Figure S6. Pore size distribution of COF-TPA-TMTA. It was derived from the nonlocal density functional theory (NLDFT) cylindrical pore mode.

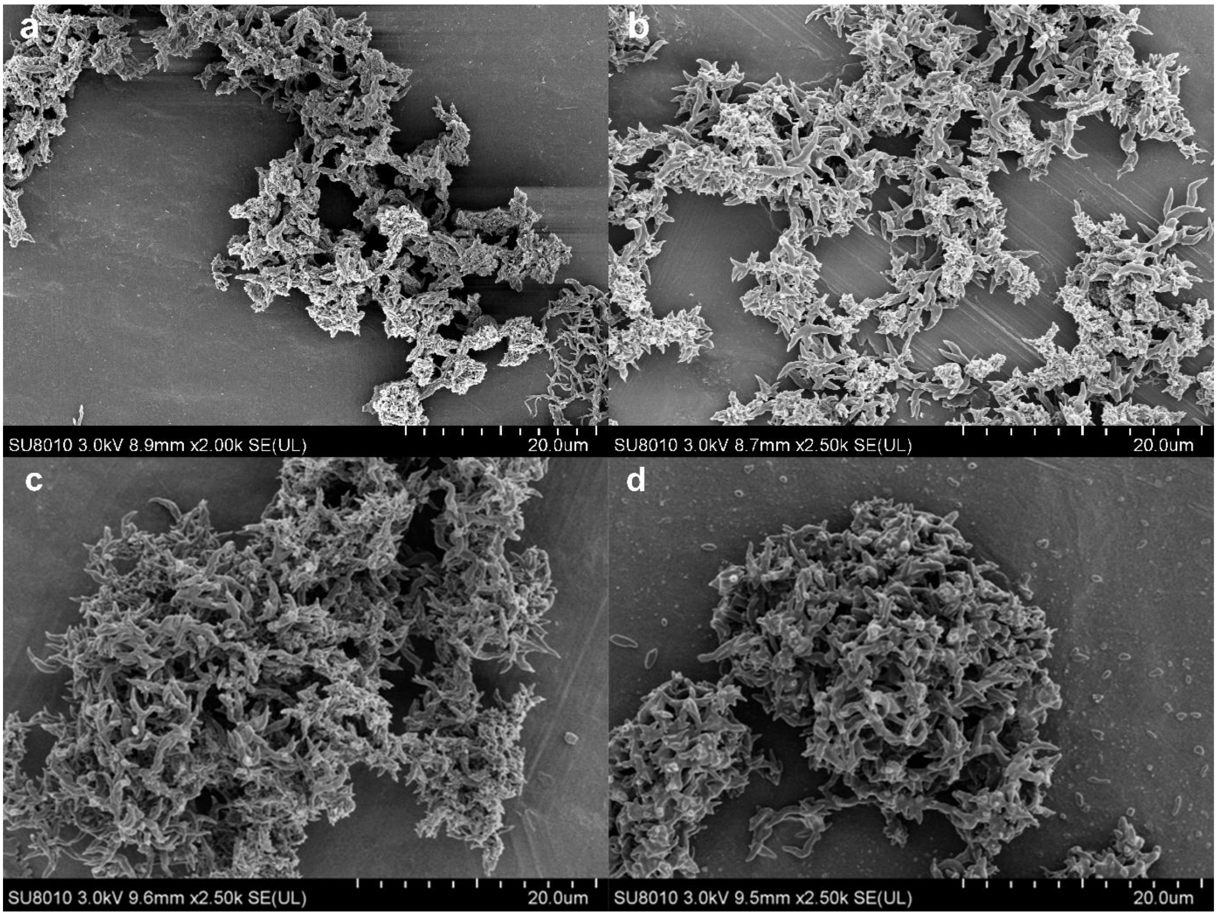

Figure S7.SEM images of COF-TPA-TMTA before (a) and after irritation (b), and COF-TPA-TMTAO (c) before and after irritation (d).Irritation conditions: 460 nm blue LED (40 W) for one week.

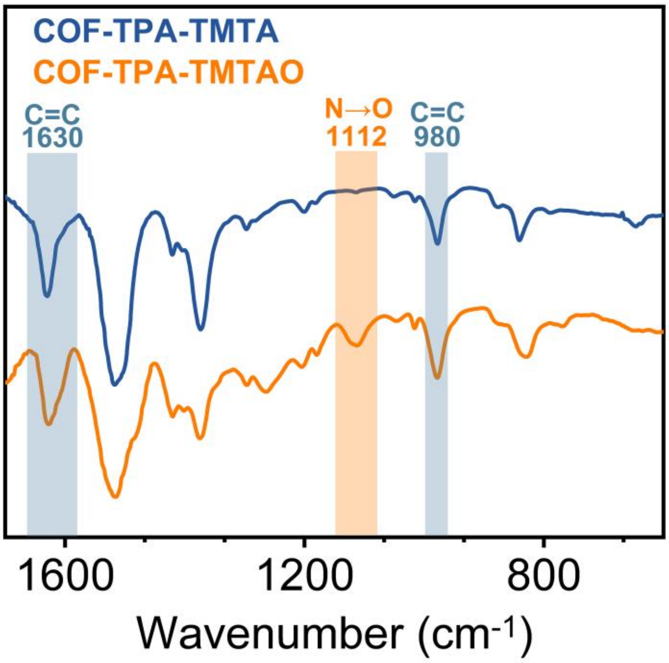

Figure S8. FT-IR spectra of COF-TPA-TMTA and COF-TPA-TMTAO.

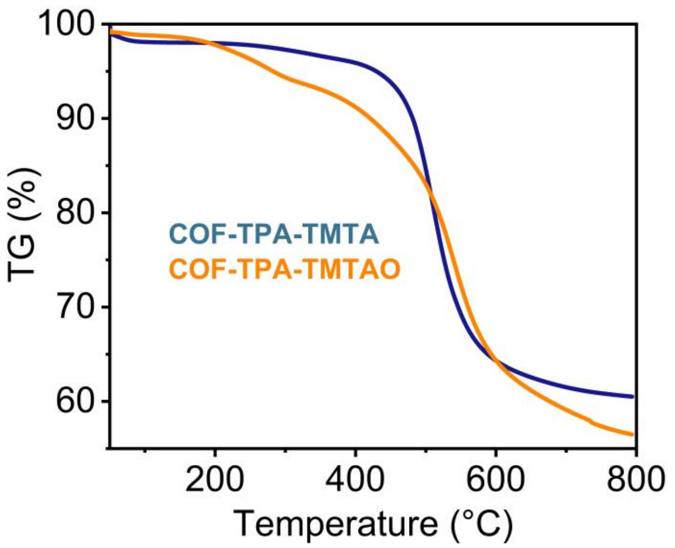

Figure S9. TG curves of COF-TPA-TMTA and COF-TPA-TMTAO.

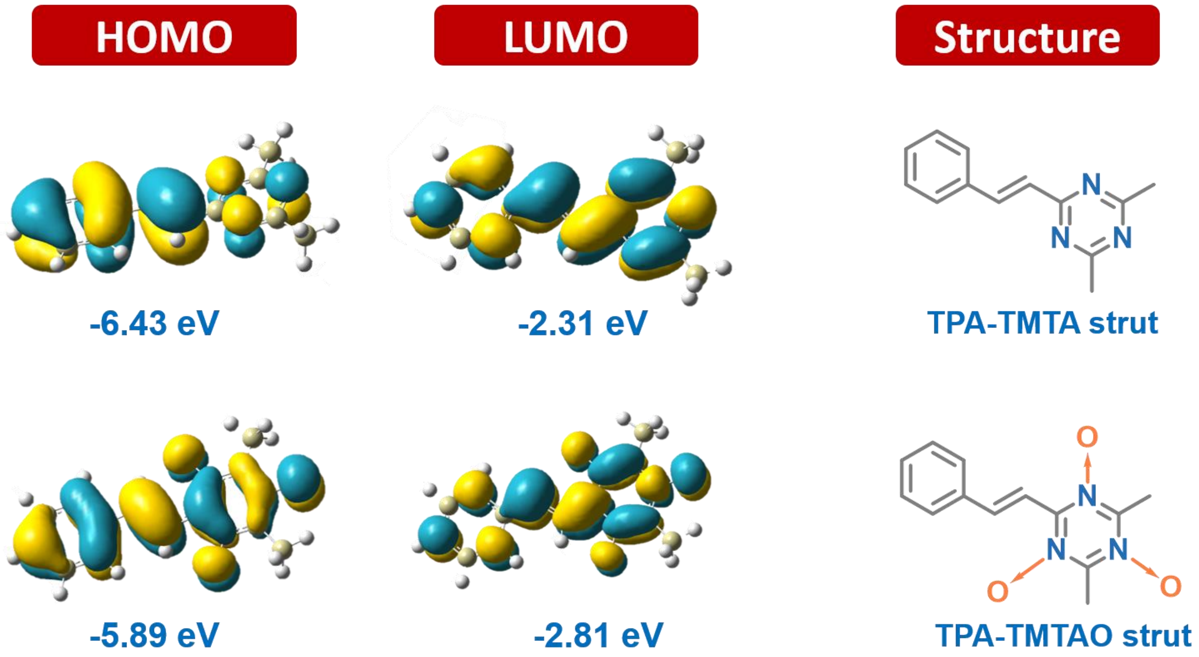

Figure S10. The natural transition orbital of COF struts.

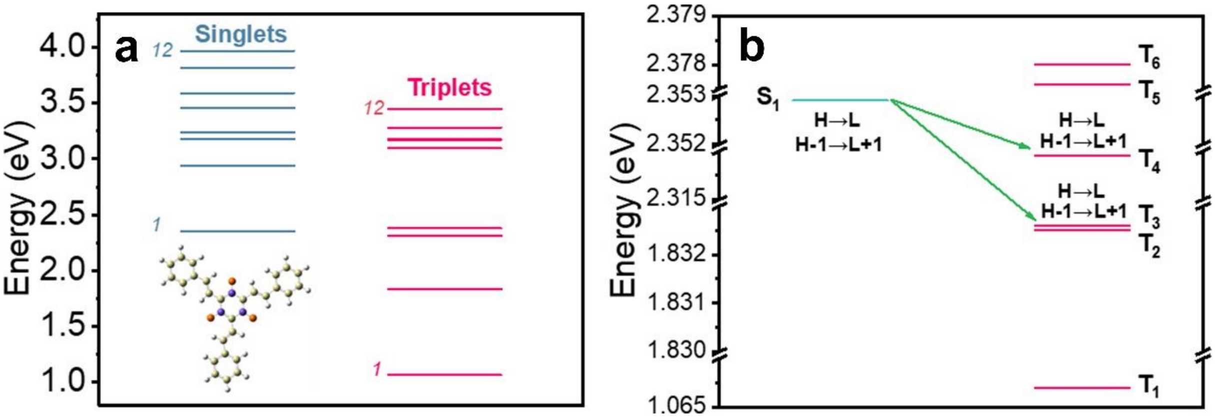

	n	Energy (eV)	Transition configuration (%)
S_n	1	2.3529	**H -> L (32.5), H-1 -> L+1 (32.4),** H-1 -> L (16.9), H -> L+1 (16.9)
T_n	1	1.0654	H -> L+1 (33.8), H-1 -> L (33.8), H -> L (17.5), H-1 -> L+1 (17.4)
	2	1.8325	H -> L+1 (27.2), H-1 -> L (27.1), H -> L (18.3), H-1 -> L+1 (18.3)
	3	1.8326	**H -> L (27.2), H-1 -> L+1 (27.1),** H-1 -> L (18.3), H -> L+1 (18.2)
	4	2.3159	**H-1 -> L+1 (32.5), H -> L (32.2),** H -> L+1 (16.8), H-1 -> L (16.8)
	5	2.3776	H-2 -> L (55.1), H-1 -> L+2 (18.3), H -> L+2 (10.3)
	6	2.3780	H-2 -> L+1 (55.0), H -> L+2 (18.3), H-1 -> L+2 (10.3)

Figure S11. (a) Kohn-Sham frontier orbital analysis, (b) calculated energy levels and possible ISC channels of different model compounds of the repeated unit of COF-TPA-TMTAO, and the singlet and triplet excited states transition configurations revealed by time-dependent densityfunctional theory (TD-DFT) calculations. The matched excited states that contain the same orbital transition components of S1 were highlighted in red.

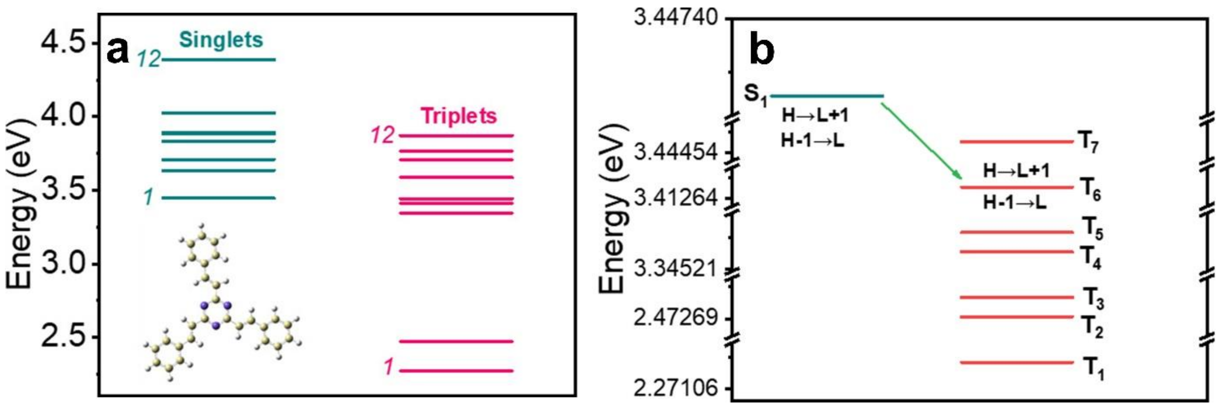

	n	Energy (eV)	Transition configuration (%)
S_n	1	3.4417	**H -> L+1 (30.5), H-1 -> L (30.4),** H -> L (18.7), H-1 -> L+1 (18.6)
T_n	1	2.2712	H -> L (25.7), H-1 -> L+1 (25.7), H-2 -> L+2 (14.6), H -> L+1 (14.2), H-1 -> L (14.1)
	2	2.4727	H-2 -> L+1 (32.1), H -> L (20.2), H-1 -> L+1 (20.2), H-1 -> L+2 (14.9)
	3	2.4728	H-2 -> L (32.1), H-1 -> L (20.1), H -> L+1 (20.1), H -> L+2 (14.9)
	4	3.3453	H-2 -> L (36.8), H -> L+1 (24.4), H-1 -> L (24.3)
	5	3.3454	H-2 -> L+1 (36.8), H -> L (24.3), H-1 -> L+1 (24.3)
	6	3.4127	**H-1 -> L (31.5), H -> L+1 (31.5),** H-1 -> L+1 17.2%, H -> L 17.1%
	7	3.4446	H-3 -> L 32.4%, H-4 -> L+1 32.0%, H-3 -> L+1 15.0%, H-4 -> L 14.8%

Figure S12. (a) Kohn-Sham frontier orbital analysis, (b) Calculated energy levels and possible ISC channels of different model compounds of the repeated unit of COF-TPA-TMTA, and the singlet and triplet excited states transition configurations revealed by TD-DFT calculations. The matched excited states that contain the same orbital transition components of S1 were highlighted in red.

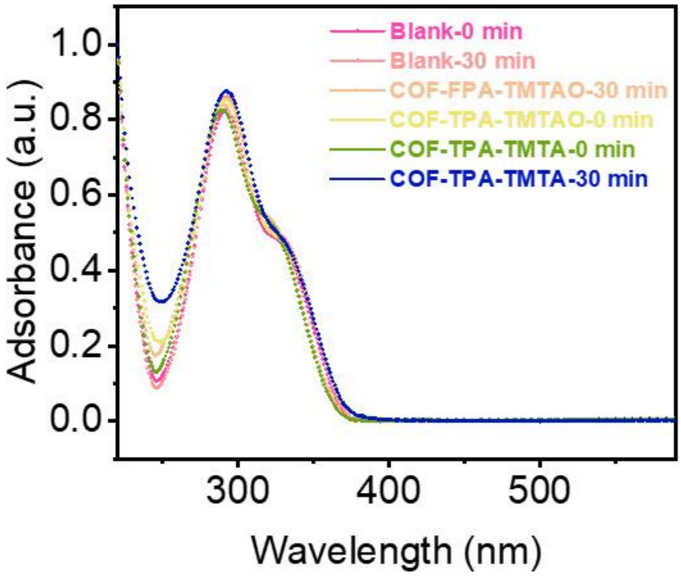

Figrue S13.UV-Vis spectra of coumarin-3-carboxylic acid under various conditions to detect the generation of •OH species. The absence of characteristic UV-Vis absorbance associated with the adduct of•OH and coumarin-3-carboxylic acid suggested that no •OH species was yielded.

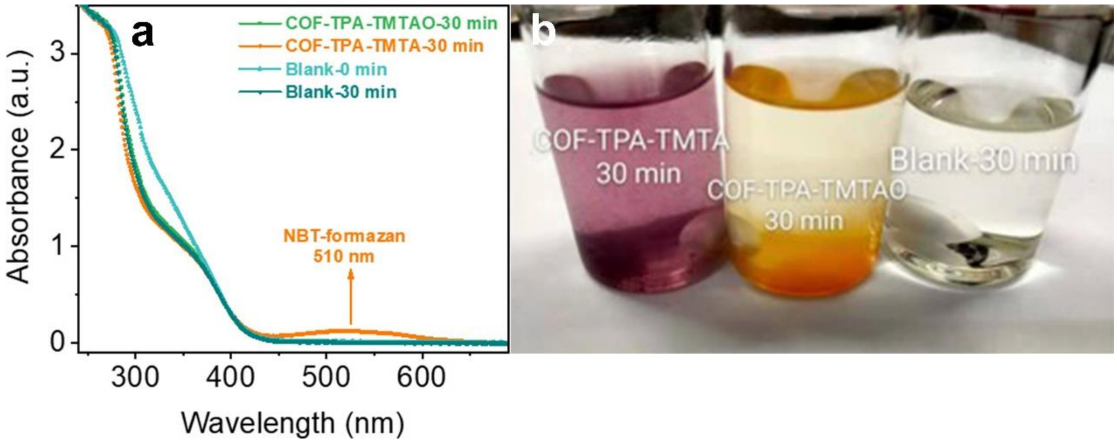

Figrue S14. (a) UV-Vis spectra of nitrotetrazolium blue chloride under various conditions to detect the generation of $O_2^{\bullet-}$ species. The appearance of characteristic UV-Vis absorbance associated with the adduct of $O_2^{\bullet-}$ and nitrotetrazolium blue chloride in the presence of COF-TPA-TMTA suggested that there were •OH species. In contrast, the absence of characteristic UV-Vis absorbance associated with the adduct of •OH and nitrotetrazolium blue chloride suggested that no •OH species was yielded in the presence of COF-TPA-TMTA or the absence of COF materials. (b) The corresponding color changes.

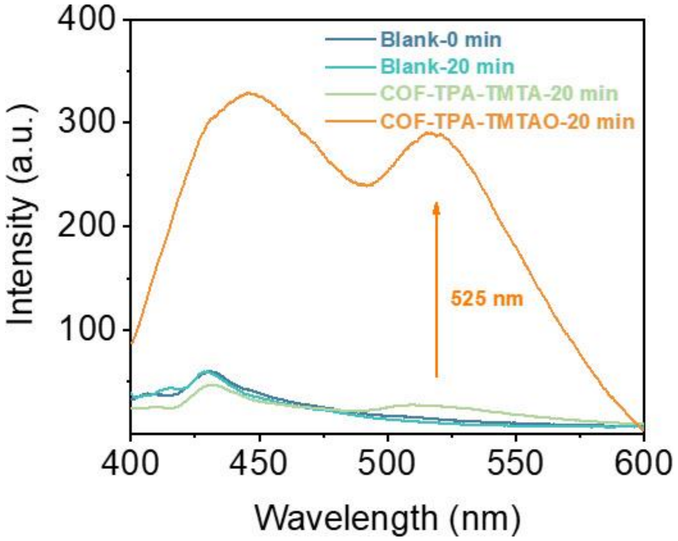

Figrue S15. Fluorescence spectra of singlet oxygen sensor green under various conditions to detect the generation of $^1O_2$ species. The appearance of characteristic Fluorescence absorbance associated with the adduct of $^1O_2$ and singlet oxygen sensor green in the presence of COF-TPA-TMTA and COF-TPA-TMTAO suggested that there were $^1O_2$ species.

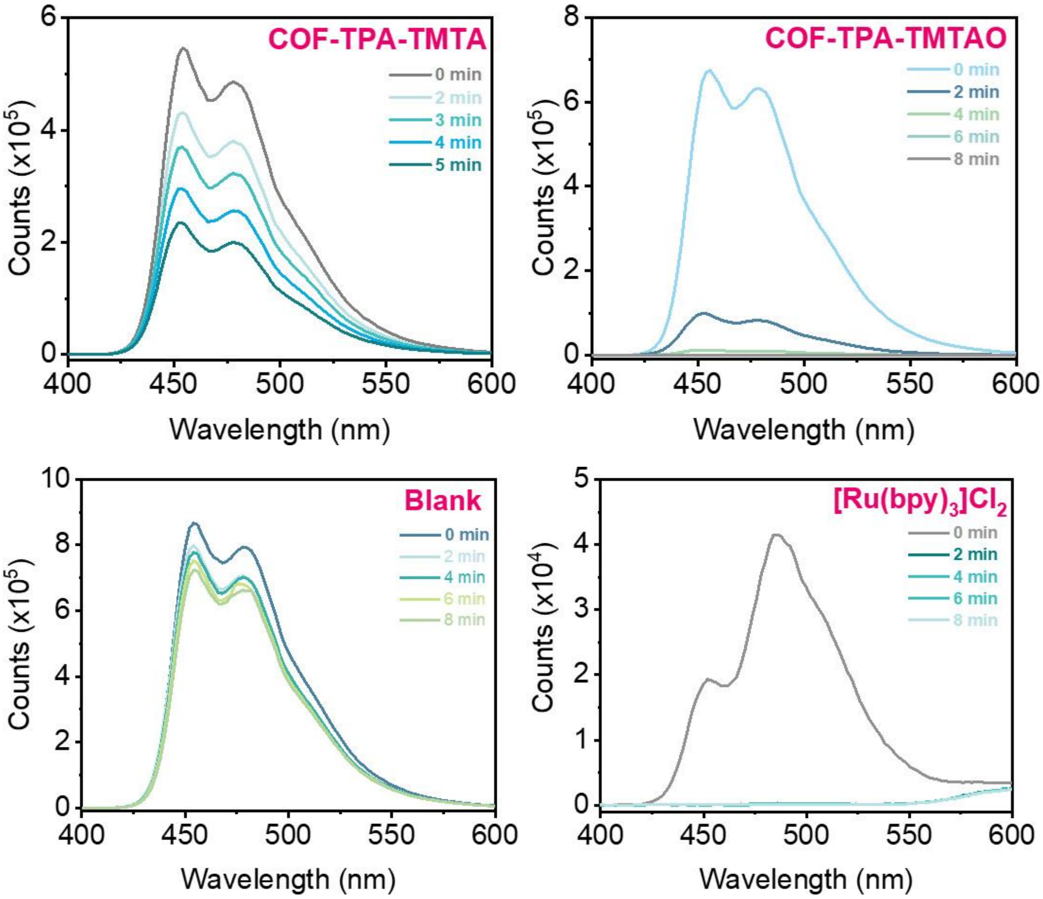

Figure S16. Time-resolved emission spectra changes of various systems in the 1,3-diphenyl-isobenzofuran under irradiation.

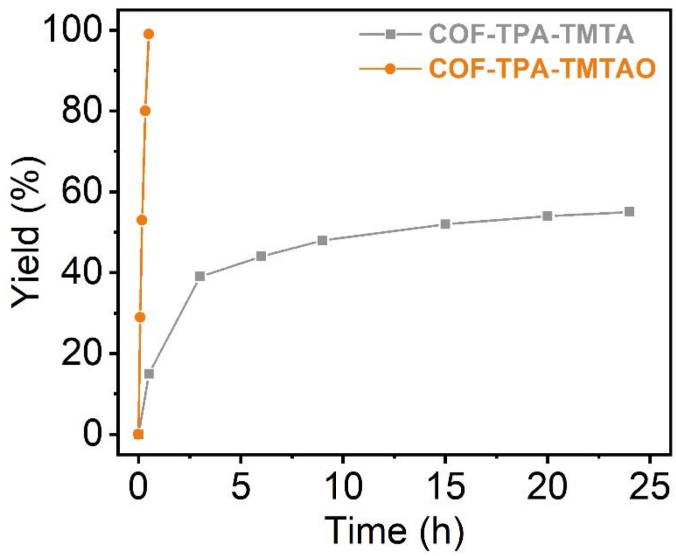

Figure S17. Plots of methyl phenyl sulfoxide yields verse time of thioanisole oxidation.

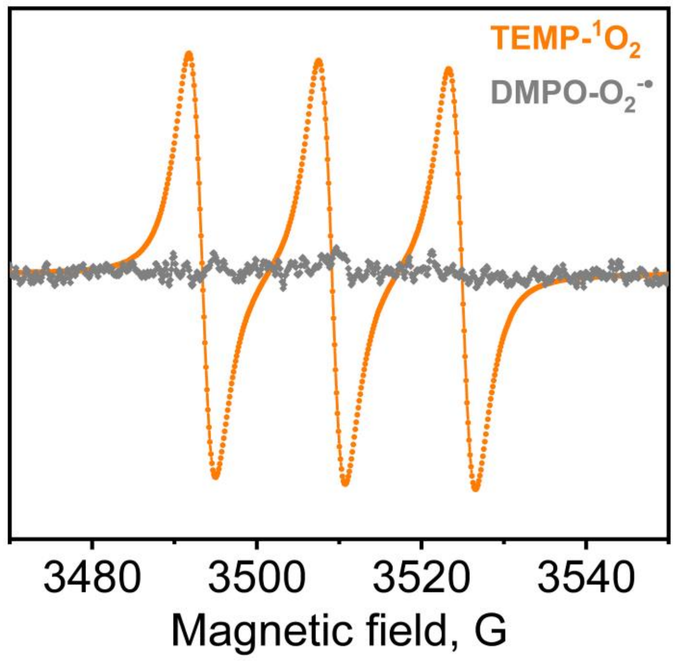

Figure S18. EPR spectra of MeOH solution of DMPO and TEMP in the presence of COF-TPA-TMTAO after irradiation under visible light.

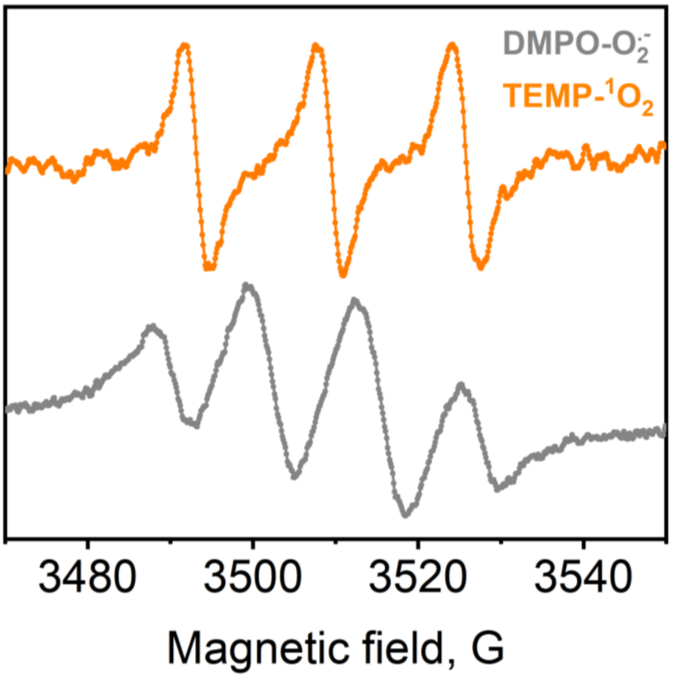

Figure S19. EPR spectra of MeOH solution of DMPO and TEMP in the presence of COF-TPA-TMTA after irradiation under visible light.

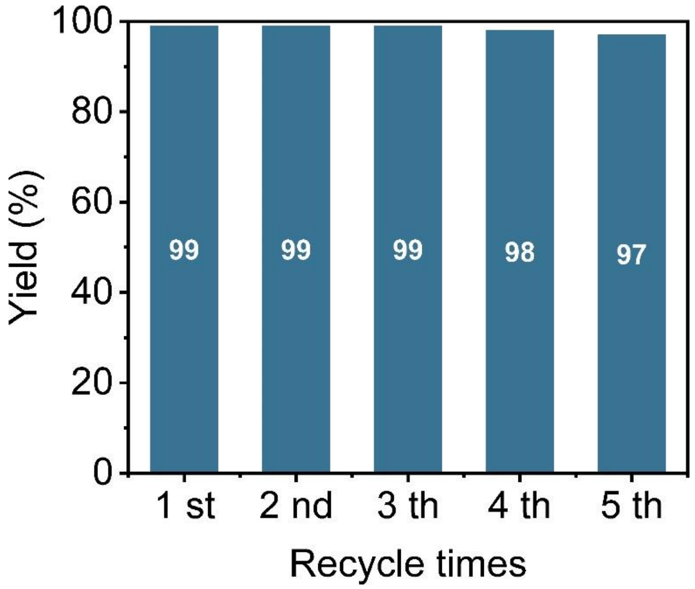

Figure S20. Recycling performance of COF-TPA-TMTAO in the transformation of thioanisole into methyl phenyl sulfoxide.

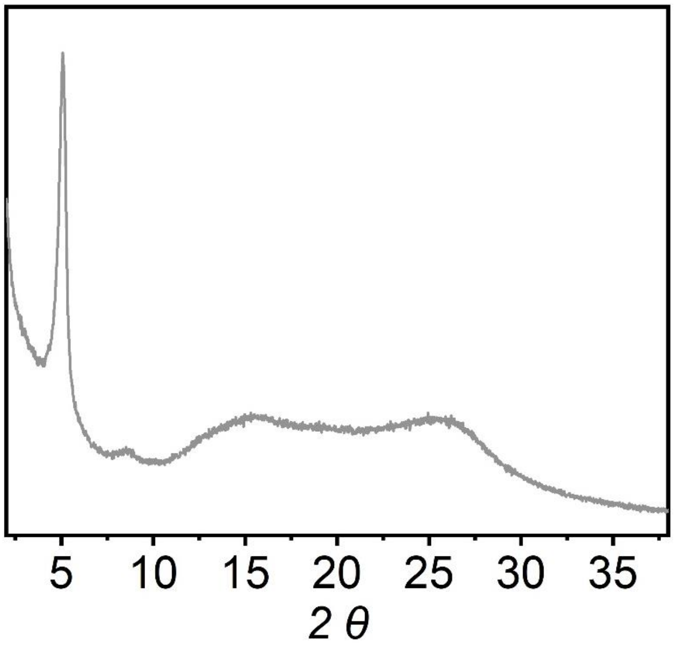

Figure S21.XRD pattern of the used COF-TPA-TMTAO.

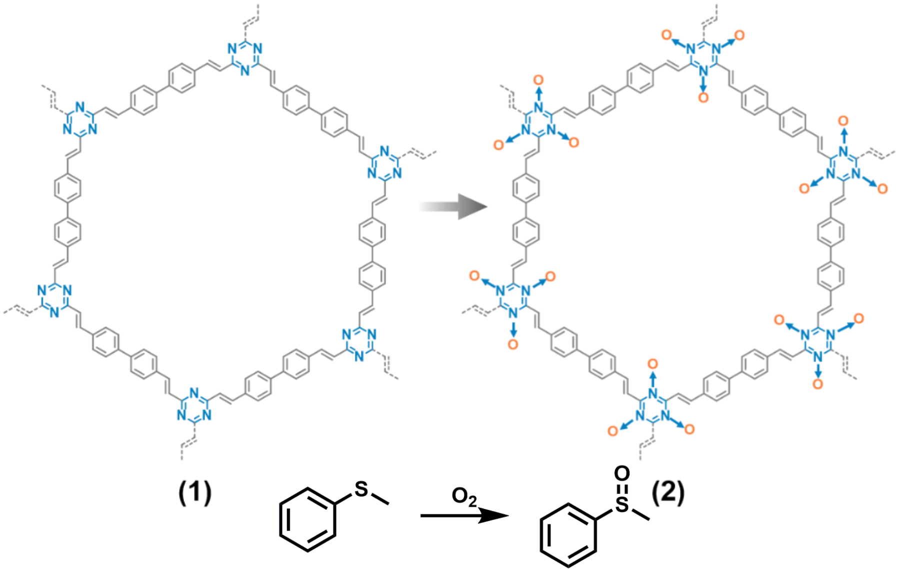

Catalyst	Yield (%)
**1**	65
**2**	99

Figure S22. Structures and catalytic performance comparison before and after oxidation. Reaction conditions: thioanisole (0.25 mmol) and COF material (10 mg) in MeOH (2 mL) at room temperature under blue LED irradiation (40 W) for 90 min.
